# Recent Advances in the Development of Functional Nucleic Acid Biosensors Based on Aptamer-Rolling Circle Amplification

**DOI:** 10.3390/molecules30112375

**Published:** 2025-05-29

**Authors:** Ce Liu, Wanchong He

**Affiliations:** 1 College of Science, Yanbian University, Yanji 133002, China; 2 Shandong Key Laboratory of Applied Technology for Protein and Peptide Drugs, School of Pharmaceutical Sciences and Food Engineering, Liaocheng University, Liaocheng 252000, China

**Keywords:** aptamer, rolling circle amplification, biosensor

## Abstract

Aptamers are synthetic nucleic acids or peptides that exhibit high specificity and affinity for target molecules such as small molecules, proteins, or cells. Due to their ability to bind precisely to these targets, aptamers have found widespread use in bioanalytical and diagnostic applications. Rolling circle amplification (RCA) is an amplification technique that utilizes DNA or RNA templates, where circular primers are extended by polymerases to generate multiple repeated sequences, enabling highly sensitive detection of target molecules. The integration of aptamers with RCA offers significant advantages, enhancing both the specificity and sensitivity of detection while ensuring a fast and straightforward process. This synergy has already been widely applied across various fields, including fluorescence, microfluidics, visualization, and electrochemical technologies. Examples include molecular probe development, rapid detection of disease biomarkers, and environmental monitoring. Looking ahead, the aptamer-RCA platform holds great promise for advancing early disease diagnosis, precision medicine, and the development of nanosensors, driving innovation and new applications in these fields.

## 1. Introduction

Functional nucleic acids (FNAs) are a class of nucleic acid molecules that exhibit a wide range of biological and chemical functions, including molecular recognition, catalysis, gene regulation, and signal amplification. For instance, aptamers function as high-affinity molecular recognition elements, while DNAzymes exhibit catalytic activity similar to that of protein enzymes. Other FNAs, such as ribozymes, regulate gene expression by cleaving target RNAs, and molecular beacons serve as signal transducers in biosensing platforms [1]. Aptamers are a type of functional nucleic acid whose structure is formed by the folding of single-stranded DNA or RNA sequences into complex three-dimensional conformations. This structure endows aptamers with high specificity, high affinity, stability, and reversibility, making them widely applicable in fields such as therapy and biosensing [2]. The ability to chemically synthesize and modify aptamers makes them a powerful alternative to antibodies. Aptamer molecules were first discovered in the 1990s by two independent laboratories, namely the Gold and Szostak groups [3]. Systematic evolution of ligands by exponential enrichment (SELEX) is the core technology for aptamer screening. Its process mainly consists of five key steps: First, construct a nucleic acid library containing a vast number of random sequences (usually 20–100 nucleotides), providing a basis for diversity in screening [4]. Then, incubate this nucleic acid library with target molecules (such as proteins, small molecules, or cells) under optimized conditions to allow specific sequences to bind to the targets [5]. Next, separate the nucleic acid molecules that bind to the targets from the unbound ones through methods like solid-phase separation, membrane filtration, or capillary electrophoresis [6]. Subsequently, amplify these bound nucleic acid sequences using PCR (for DNA libraries) or reverse transcription-PCR (for RNA libraries) [7]. Finally, repeat this screening process 8–15 times and gradually increase the stringency of the screening to enrich aptamer sequences with high affinity and high specificity [8]. The development of aptamers, from their discovery to their applications, has progressed rapidly. Advances in SELEX technology and interdisciplinary research have unlocked their immense potential in therapy, biosensing, nanotechnology, and basic research. Despite some challenges, with ongoing technological advancements, aptamers are expected to play an even more significant role in the future of precision medicine and biotechnology [9].

Rolling circle amplification (RCA) is a highly efficient nucleic acid amplification technique that utilizes a circular DNA template to perform isothermal amplification under the action of DNA polymerase, generating a linear single-stranded DNA product containing multiple repeating units. The RCA reaction consists of three core components: a circular DNA template serves as the replication backbone, a primer provides the starting site, and a DNA polymerase (such as Phi29) catalyzes the extension at a constant temperature. After the primer binds to the circular template, the polymerase continuously replicates in a cycle, generating a long-chain DNA containing repeating units. This system can integrate aptamer sequences by designing the template to achieve highly sensitive detection of target molecules, and it is widely used in the field of biosensing [10]. The RCA reaction is characterized by isothermal amplification, high sensitivity, high specificity, and multifunctionality. The characteristics of the RCA reaction are related to the substances that make up the reaction. The material basis of the isothermal reaction is that the temperature at which the reaction occurs is the same as the optimal temperature of the enzyme [11]. The reaction is highly sensitive because it can undergo numerous elongations and amplifications, achieving the corresponding signal amplification in detection. The stability, speed, and sensitivity of the RCA strategy have enabled its widespread application in DNA and protein detection, single nucleotide polymorphism analysis, in situ signal amplification, drug discovery, and nanotechnology [12].

This review introduces the application of RCA combined with aptamers in biosensing detection. Additionally, it points out that this combination offers significant advantages, making it an important tool for highly sensitive and specific detection, and explains that aptamers, as molecular recognition elements, can specifically bind to target molecules (such as proteins [13], small molecules [14], or cells [15]), while the RCA reaction significantly amplifies the binding signal through an isothermal amplification mechanism. This review also shows that the combined strategy not only improves detection sensitivity but also enables accurate detection of trace targets in complex samples, and it further mentions that the versatility of the rolling circle amplification product makes it compatible with a variety of detection methods, such as fluorescence [16], colorimetry [17], and electrochemistry [18], thus making it suitable for fields such as disease diagnosis [19], environmental monitoring and food safety [20]. Furthermore, it predicts that in the future, with the further optimization of aptamer screening technology and the rolling circle amplification reaction system, this strategy will play a greater role in point-of-care testing and precision medicine [21].

## 2. Aptamer

Aptamers are short, single-stranded DNA or RNA oligonucleotides (typically 15–80 nucleotides) capable of folding into stable, unique tertiary structures. These structures facilitate high-affinity and specific binding to diverse targets through non-covalent interactions such as hydrogen bonding and π–π stacking [22]. Due to their synthetic origin and chemical versatility, aptamers function as effective molecular recognition elements across a broad range of biosensing applications [23].

Their key features—including high specificity [23], stability [24], and ease of synthesis—underpin their growing role in diagnostics and therapeutics. Unlike antibodies, aptamers can be chemically synthesized and modified, which allows for scalable, cost-effective production with high batch-to-batch consistency [24]. The conventional SELEX method is widely used for aptamer selection but is often time-consuming. Recent optimization strategies have significantly improved screening efficiency. For instance, Yang et al. used capillary electrophoresis-SELEX (CE-SELEX) to isolate a high-affinity aptamer for human apotransferrin within only three rounds [25]. Similarly, Liu et al. developed a microfluidic-based protein microarray system–SELEX (PMM-SELEX), achieving reproducible aptamer selection with high throughput and precision [26].

Aptamers are now being developed for use in neurological and cancer therapies, pathogen diagnostics, and precision drug delivery [27]. Nonetheless, challenges such as rapid renal clearance, structural instability, and potential off-target effects hinder their full therapeutic potential. Ongoing research in chemical modification and delivery systems is vital to overcoming these limitations and enhancing their applicability in clinical settings [28].

## 3. The Regulation of RCA Reaction

By regulating the accessibility of at least one of the four essential components of RCA, the initiation of the RCA reaction can be controlled. These four essential components are as follows: the primer, the circular DNA template (CDT), the DNA polymerase (usually φ29 DNA polymerase unless otherwise specified), and deoxyribonucleoside triphosphates (dNTPs). To date, there have been no reported methods for regulating the RCA reaction by modulating the accessibility of the DNA polymerase or dNTPs. Here, we focus on the techniques specifically designed to regulate the accessibility of the primer strand or the circular DNA template.

### 3.1. The Design of Primers

#### 3.1.1. The Primer Is Directly Connected to the Aptamer

In an early example, some scientists utilized a thrombin-binding structure-switching aptamer to regulate the RCA reaction through target-induced primer displacement [29]. In this case, the primer was first captured by an immobilized DNA probe at its 5′ end. After performing a washing step, the CDT and φ29 DNA polymerase were added to the captured primer, enabling the generation of target-mediated rolling circle amplification products from the 3′ end.

**Figure 1:**
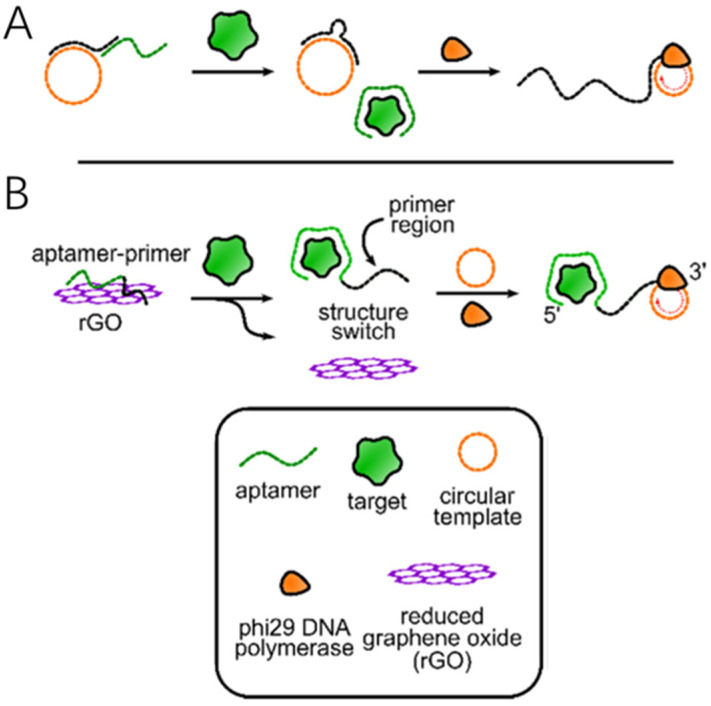
Structure switching primer design strategies: (**A**) Tripartite structure-switching. (**B**) Structure switching using reduced graphene oxide material [30].

Over the past few years, Bialy, RM et al. designed a method that combines the primer and the aptamer without the need for an immobilized DNA probe or a washing step [30]. In this approach, the 5′-end of the primer is hybridized with the circular DNA template, and the other 3′-end is bound to the aptamer. In the absence of the target substance, the aptamer prevents the primer from binding to the template; thus, the rolling circle amplification reaction cannot be initiated. When the target substance is added to the system, the aptamer is released and binds to the target substance. The 3′ end of the primer successfully binds to the circular template, forming a complementary primer-template double strand [31]. Under the catalytic action of φ29 DNA polymerase, the RCA reaction proceeds smoothly (Figure 1A).

However, to ensure the smooth progress of the RCA reaction, the aptamer must be smoothly released upon target binding, and at the same time, the primer should have a certain affinity for the circular DNA template. To achieve these two points, it is important to introduce a new substance that can both immobilize the aptamer and make it easily detachable. In 2009, scientists proposed using graphene oxide to adsorb the primer–aptamer complex. Graphene oxide has adjustable properties that enable it to retain single-stranded or double-stranded DNA on its surface. When the aptamer binds to the target substance to form a complex, the aptamer adsorbed on the surface of the graphene oxide will detach, thus initiating the rolling circle amplification reaction (Figure 1B). According to a reported multifunctional biosensing platform, reduced graphene oxide can non-specifically adsorb single-stranded DNA molecules, and DNA aptamers can bind to reduced graphene oxide. The effect of reduced graphene oxide in the RCA reaction is better than that of graphene oxide [32].

#### 3.1.2. RNA-Cleaving DNAzyme (RCD) Mediated Primer Regulation

RCD is a class of functional DNA molecules with RNA cleavage activity. It can specifically recognize and cleave RNA substrates just like protein ribonuclease [33] and has important applications in the fields of biotechnology and medicine. It can be used in combination with the RCA reaction. In the RCA reaction, RCD can cleave the RNA sequences in the RCA products to release reporter molecules. It can also initiate downstream reactions to release a new set of RCA primers, enabling multiple rounds of signal amplification. Several strategies have been developed to regulate primers in the RCA reaction mediated by RCD.

##### (1) RCD Cleavage for Primer Release

In the RCA reaction, the primer precursor can be designed to contain the specific cleavage site of RCD (such as an RNA sequence or an RNA-DNA hybrid strand). In the initial state, the primer is in a closed or inactive state. When the target (such as a specific RNA or metal ion) activates the RCD, the RCD cleaves the primer precursor, releasing an active primer with a 3′-OH end, thus initiating the RCA amplification. This method is highly target-dependent and can effectively avoid non-specific amplification, making it suitable for the highly sensitive detection of low-abundance RNAs (such as miRNA or viral RNA). For example, in nucleic acid detection, after the target RNA activates the RCD, it cleaves the primer precursor to release the functional primer, which then binds to the circular template for RCA, achieving signal amplification.

##### (2) RCD Regulation of Primer Conformation

By designing the primer as a hairpin structure or a molecular beacon and embedding the cleavage site of RCD (such as an RNA sequence) in its stem, the dynamic regulation of primer activity can be achieved. In the unactivated state, the primer cannot bind to the template due to the closure of its secondary structure. When the RCD is activated by the target, it cleaves the hairpin stem, causing the primer conformation to change from a closed state to a linear structure, exposing the sequence complementary to the circular template and thus initiating the RCA. This regulation method can significantly reduce the background signal and improve the signal-to-noise ratio of the detection, especially suitable for RNA imaging in living cells or real-time monitoring. For example, in fluorescence detection, after the RCD cleaves the hairpin primer, the fluorescent group and the quenching group are separated, and at the same time, the active primer is released to trigger the RCA, achieving double signal amplification.

##### (3) RCD-Mediated Primer Generation

The RCD can directly generate new RCA primers by cleaving long-chain RNAs or DNA-RNA hybrid strands instead of relying on pre-designed primer precursors. When the target is present, the RCD recognizes and cleaves a specific sequence, and the generated short fragment (such as a DNA containing a 3′-OH end) can serve as a functional primer to initiate the RCA. This method is suitable for complex samples (such as cell lysates or clinical samples) and can achieve exponential signal amplification through cascade reactions. For example, in the detection of viral RNA, the target hybridizes with the auxiliary probe to form the cleavage site of the RCD. After the RCD cleaves it, a short DNA primer is generated, which in turn triggers the RCA amplification. Combined with fluorescent or electrochemical signal output, highly sensitive detection can be achieved.

##### (4) RCD Regulation of Primer Competition

By introducing competitive primers (containing the cleavage site of RCD) to coexist with functional primers, the dynamic regulation of RCA can be achieved. The competitive primers preferentially bind to the circular template but do not extend, thus inhibiting the function of the functional primers. When the RCD is activated by the target, it cleaves the competitive primers to make them dissociate, release the closure of the template, and enable the functional primers to dominate the RCA reaction. This strategy can be used to construct logical gating systems (such as AND/OR gates) to achieve the coordinated detection of multiple targets. For example, in nucleic acid computing, two targets, respectively, activate different RCDs. After cleaving the corresponding competitive primers, the functional primers initiate the RCA and output specific signals.

##### (5) RCD Control of Primer Recycling

The RCD can continuously cleave the primer complementary sequence in the RCA extension product, releasing the bound primer and allowing it to participate in the amplification cycle again. This method can significantly improve the utilization rate of primers and achieve continuous signal amplification, which is suitable for the detection of ultra-low concentration targets. For example, multiple RCD cleavage sites are embedded in the long-chain RCA product. The RCD repeatedly cleaves to release the primer, initiating multiple rounds of amplification. Combined with fluorescent or nanoparticle signal output, the detection sensitivity can be improved to the single-molecule level.

### 3.2. The Design of Circular Template

#### 3.2.1. Ligate the Circular Template

The principle of constructing circular templates mediated by T4 DNA ligase is based on the enzymes’ ability to catalyze the formation of a phosphodiester bond between the 5′-phosphate group and the 3′-hydroxyl group of DNA molecules, thereby achieving the circular ligation of linear DNA molecules [34]. This method is applicable to both single-stranded and double-stranded DNA templates. For single-stranded DNA, the 5′ end needs to be phosphorylated in advance, while double-stranded DNA needs to be denatured into single-strands before use. There are two types of ligation methods: high-efficiency (>90%) sticky-end ligation and low-efficiency (about 20%) blunt-end ligation. The former requires the design of complementary overhanging end sequences. The characteristics of this technology include mild reaction conditions (usually carried out at 16 °C) and good stability of the ligation products. It is particularly suitable for the circularization of DNA in the length range of 50–1000 nt. However, it has limitations, such as low blunt-end ligation efficiency and the tendency to produce multimer by-products. The circularization efficiency can be improved by optimizing reaction conditions (e.g., adding PEG8000 and controlling DNA concentration).

#### 3.2.2. Design Circular Aptamers

Circular aptamers play multiple crucial roles in the RCA reaction. Their unique structural characteristics significantly enhance the amplification efficiency and detection performance [35]. As a core component of the circular template, circular aptamers optimize the RCA reaction through the following mechanisms: First, their closed-loop structure eliminates the end-inhibition effect, enabling DNA polymerase to perform continuous and efficient strand-displacement synthesis, which boosts the amplification efficiency by 10–100 times. Second, the aptamer sequence can specifically bind to target molecules (such as proteins, small molecules, or cell markers). In the reaction, it serves as both a recognition element and a signal transducer, achieving integrated “recognition–amplification” detection. For example, in thrombin detection, the circular aptamer can specifically bind to the target protein and also act as an RCA template to guide the synthesis of repetitive sequences [36]. Through cascade amplification, a single binding event is converted into thousands of copies of nucleic acid signals. In addition, circular aptamers can integrate functional sequences (such as promoters, ribozyme sites, or fluorescent labels) through modular design to achieve multiplex detection or intelligent regulation. Compared with linear aptamers, the circular structure also significantly improves the resistance to nucleases, allowing the reaction to remain stable in complex biological samples. These characteristics have led to the widespread application of RCA technology based on circular aptamers in fields such as ultrasensitive biosensing, single-cell analysis, and in vitro diagnostics, with a detection limit reaching the fM or even aM level. The latest research has also developed dynamic response systems through engineered circular aptamers, such as allosteric aptamers sensitive to pH or temperature, further expanding the application prospects of RCA in in vivo monitoring and intelligent diagnosis.

The inherent circular structure of aptamers also enables their integration into intramolecular structure-switching systems for regulating rolling products. Zhao et al. transformed a linear aptamer targeting lipopolysaccharides into an aptamer by incorporating the aptamer and a primer-binding region within a CDT. In the absence of the target, the aptamer formed a dumbbell-shaped structure through intramolecular hybridization, which restricted access to the primer-binding region. When lipopolysaccharides were introduced, the aptamer underwent a conformational change, adopting an extended shape that exposed the primer-binding region, thereby facilitating primer binding and rolling product generation in a single-pot assay.

Roger’s research team developed a structural switching system using circular aptamers, where the binding of platelet-derived growth factor induces the detachment of the circular platelet-derived growth factor aptamer from reduced graphene oxide [30]. Once detached, the complex formed between the circular aptamer and platelet-derived growth factor is separated from reduced graphene oxide via centrifugation. The supernatant containing the released circular aptamer is then mixed with primers, enabling the generation of RCA products. A limitation of this method is the need for a centrifugation step to remove the reduced graphene oxide, as reduced graphene oxide can adsorb linear primers, thereby hindering the RCA reaction.

Another distinctive approach involves incubating a circular aptamer for glutamate dehydrogenase with magnetic beads coated with recombinant glutamate dehydrogenase to form a circular aptamer–recombinant glutamate dehydrogenase complex [30]. The addition of natural glutamate dehydrogenase causes the circular aptamer to be released from the magnetic beads. After removing the beads, the released circular aptamer can bind to the added primers, enabling an RCA reaction mediated by the natural glutamate dehydrogenase.

Most of the examples mentioned above require a separation step, which presents a significant challenge for their implementation in point-of-care detection. To overcome this issue, a method has been developed that utilizes a CDT integrated with an aptamer to regulate the RCA reaction by obstructing the ability of Phi29 DNA polymerase to transcribe the circular DNA template upon target binding. When a platelet-derived growth factor aptamer is integrated into the circular DNA template, it was observed that upon binding of the target protein, Phi29 DNA polymerase is unable to displace the bound protein, thus preventing it from transcribing the aptamer sequence and inhibiting the RCA reaction [37]. This approach eliminates the need for a separation step, simplifying the detection process considerably.

## 4. The Amplification Methods of the RCA Reaction

In RCA technology, there are two primary amplification modes—linear and exponential-distinguished by their underlying amplification mechanisms and efficiencies. These two modes exhibit significant differences in both reaction pathways and applicable scenarios.

### 4.1. Linear RCA

**Figure 2:**
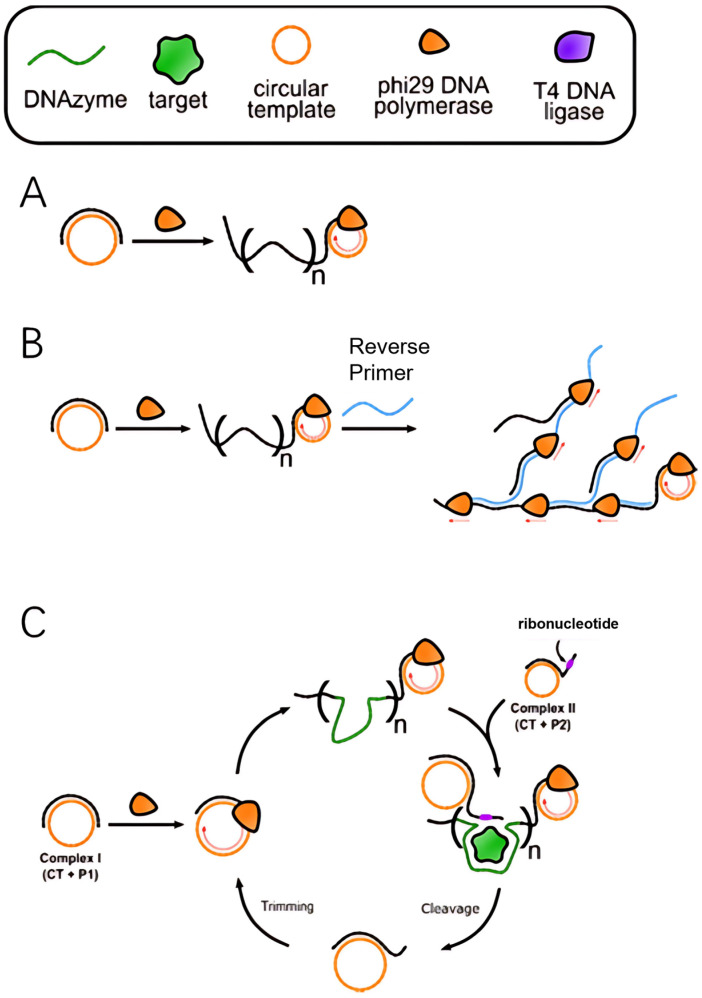
Schematic illustrations for linear and exponential amplification of RCA: (**A**) Linear RCA. (**B**) Hyperbranched RCA. (**C**) DNAzyme feedback amplification [30].

Linear RCA is the most basic form of amplification, where the core mechanism involves continuous rolling synthesis by a DNA polymerase on a circular template, generating long single-stranded DNA products containing hundreds to thousands of repeat units (Figure 2A). This process is characterized by three typical features: (1) each circular template can only produce a single continuous multi-copy product, with amplification efficiency linearly correlated with reaction time; (2) no additional primers or enzymes are required, making the reaction system simple; (3) the typical signal amplification factor ranges from 10^3^ to 10^4^. Linear RCA is particularly suited for scenarios that require stable and controlled amplification, such as in situ hybridization and protein detection. For instance, in immuno RCA technology, linear amplification is initiated by primers conjugated to antibodies, enabling the visual detection of individual antigen molecules.

### 4.2. Exponential RCA

Exponential RCA achieves signal amplification through the introduction of a multi-stage amplification mechanism, which can be implemented in two main ways:

(1) Hyperbranched RCA: Reverse primer sites are designed on the initial RCA products, and cascade amplification mediated by multiple primers leads to the formation of a three-dimensional branched structure (Figure 2B). This method can increase the amplification factor to 10^6^–10^9^ times, but precise control of primer concentration is necessary to avoid non-specific amplification [38].
(2) Primer regeneration RCA^38^: Nucleic acid endonucleases, such as Nickase or RCD, cyclically cleave RCA products, releasing new primers that initiate secondary amplification cycles (Figure 2C). This dynamic regulation approach is particularly useful for real-time quantitative detection, such as the ultrasensitive analysis of viral nucleic acids.

## 5. Applications of the Combination of Aptamers and RCA

The integration of aptamers with RCA technology has become a prominent approach in molecular diagnostics and biosensing. This strategy is particularly valuable due to its ability to combine the exceptional specificity of aptamers with the enhanced signal amplification offered by RCA. Key applications of this synergistic combination encompass (1) fluorescence-based detection, (2) microfluidic systems, (3) imaging methods, and (4) electrochemical sensing techniques.

### 5.1. Fluorescence-Based Detection

Zhu et al. designed an aptasensor utilizing a novel form of exponential rolling circle amplification for the label-free and highly sensitive fluorescence detection of ochratoxin A (OTA) [16]. G-quadruplex (G4) refers to a well-ordered, four-stranded DNA structure that forms from a single-stranded, G-rich nucleic acid sequence facilitated by the presence of K^+^ and Na^+^ [39].

**Figure 3:**
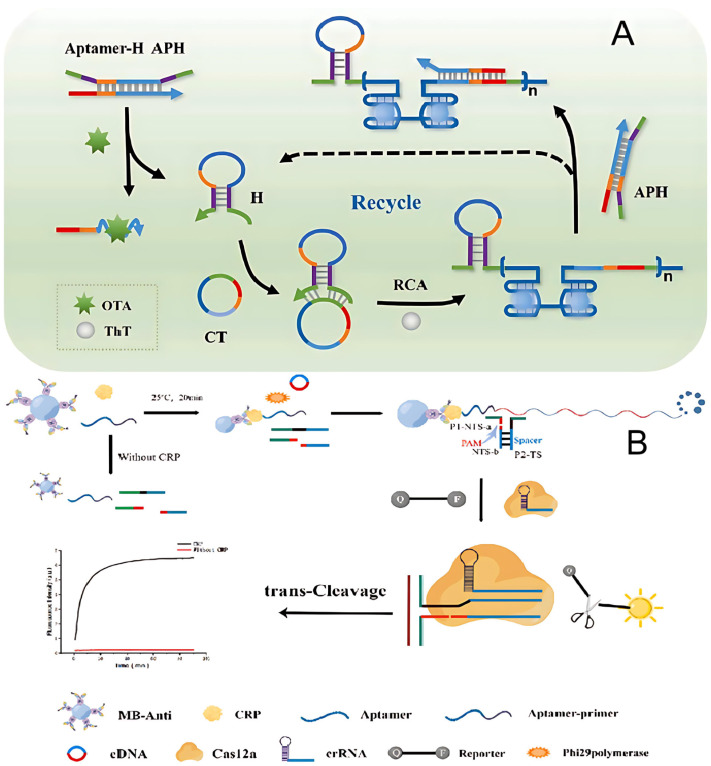
(**A**) Illustration of the fabrication of an aptasensor based on an RCA fluorescence biosensor for OTA detection. H probe was released in the presence of OTA to combine with CT to trigger RCA reaction to produce a large number of G4 signal structures (ochratoxin A (OTA), hairpin (H), circle template (CT), Thioflavin T (ThT)) [16]. (**B**) The principle of C-reactive protein assay based on RCA and CRISPR-Cas12a system dual signal amplification strategy (magnetic bead–antibody (MB-Anti), C-reactive protein (CRP)) [40].

The approach involved a circular amplification template and a multifunctional double-stranded probe, aptamer probe–hairpin primer probe (APH). APH was formed by hybridizing an aptamer probe with a hairpin primer probe in a 1:1 ratio. The aptamer probe, consisting of 51 nucleotides, included a 36-nucleotide aptamer sequence at the 3′ end that specifically recognized OTA and a 27-nucleotide region at the 5′ end designed to hybridize with RCA amplification products. The hairpin primer probe, with a stem length of 9 nucleotides, contained a 24-nucleotide loop region that was complementary to the central part of the aptamer probe, along with a 12-nucleotide primer sequence split into two segments: nine nucleotides at the 5′ end and three nucleotides at the 3′ end. These sequences were responsible for recognizing and hybridizing with the circle template, thereby triggering the RCA reaction. In the absence of OTA, the APH probe remained stable, preventing the initiation of RCA. However, when OTA was present, it bound tightly to the aptamer region, leading to the release of the hairpin probe. This release induced a conformational change in the hairpin probe, transforming it into a hairpin structure, which then hybridized with the circle template, initiating RCA. The RCA process produced amplicons containing repetitive G4 dimer structures and antisense feedback sequences. The antisense sequences interacted with the APH complex, replacing the old hairpin probe with a new one via toehold strand displacement, thus enabling the continuous initiation of RCA and the exponential amplification of the products. Simultaneously, the G4 structures formed on the RCA amplicons interacted with thioflavin T, resulting in significantly enhanced fluorescence due to the high quantum yield of the fluorescence signal (Figure 3A). This system allowed for label-free, highly sensitive detection of OTA by measuring the fluorescence intensity of the G4 dimer/thioflavin T complex [16].

Wang et al. developed a sandwich-type detection strategy that combines antibody-conjugated magnetic beads and aptamers, utilizing RCA and the nicked PAM/CRISPR-Cas12a system as signal transduction mechanisms for highly sensitive C-reactive protein detection (Figure 3B). Upon the introduction of C-reactive protein, the aptamer–primer probe bound to C-reactive protein and was subsequently captured by the antibody-conjugated magnetic beads, forming a sandwich-like complex. This complex then triggered the RCA reaction, amplifying the signal. As a result, the RCA products activated CRISPR-Cas12a trans-cleavage activity, which in turn generated a fluorescent signal [40].

**Figure 4:**
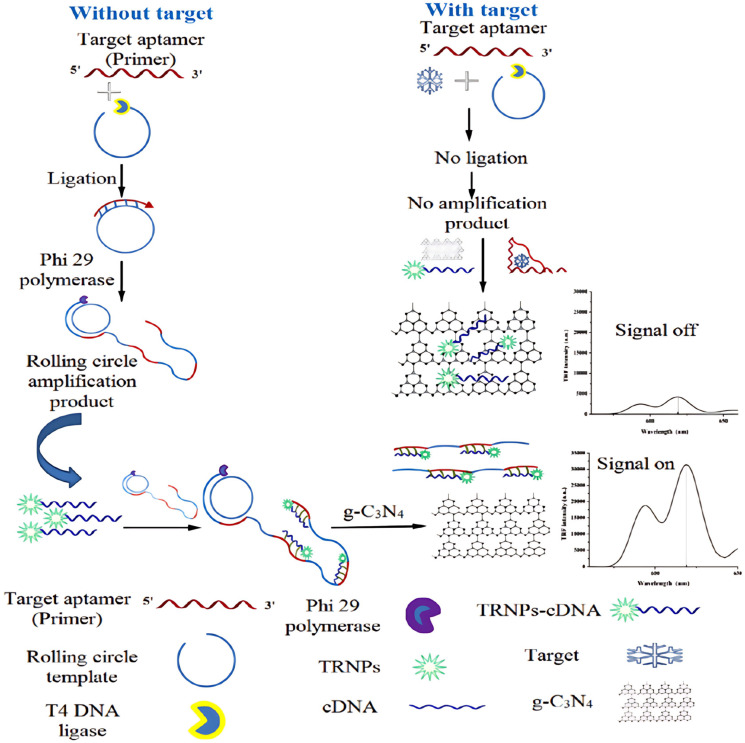
Schematic design of aflatoxin M1 detection using g-C_3_N_4_ nanosheet and time-resolved fluorescence nanoparticles combined with RCA-based DNA amplification. In the absence of aflatoxin M1, the RCA reaction was triggered by target aptamer (primer) to dismantle cDNA on the g-C_3_N_4_ surface and produce a fluorescence signal finally (time-resolved fluorescence nanoparticles (TRFNPs)) [41].

Niazi et al. developed a fluorescent aptasensor based on RCA and the fluorescence quenching effect of g-C_3_N_4_ nanosheets for the detection of aflatoxin M1. The aptamer, serving as a primer, initiates RCA in the presence of the target. In the absence of aflatoxin M1, the RCA template hybridizes with the aptamer, forming a circular DNA that undergoes circularization via T4 DNA ligase. This circular DNA then triggers RCA, facilitated by phi29 DNA polymerase, producing a long ssDNA product with multiple copies of the target aptamer sequence. The RCA product is labeled with complementary DNA, which acts as a signal probe for target detection. This circular DNA binds to multiple sites on the RCA product to form a duplex with time-resolved fluorescence nanoparticles (TRFNPs). When no target is present, the RCA product/TRFNPs-circular DNA duplex cannot bind to the g-C_3_N_4_ nanosheet due to the stronger affinity of g-C_3_N_4_ for ssDNA over dsDNA, meaning the fluorescence of the probe remains unquenched (signal on). However, when aflatoxin M1 is present, the aptamer preferentially binds to the target, inhibiting the hybridization of the aptamer with RCT and halting RCA. As a result, no RCA product is formed, and the free circle DNA is adsorbed onto g-C_3_N_4_, leading to fluorescence quenching. The extent of fluorescence quenching increases with the target concentration, as more aptamers bind to aflatoxin M1, preventing hybridization with RCA product and increasing the amount of free circle DNA (Figure 4). The strong interaction between g-C_3_N_4_ and ssDNA quenches the fluorescence via static quenching, allowing for quantitative measurement of the target by monitoring the fluorescence intensity after adding g-C_3_N_4_ [41].

Zhang et al. developed a method where streptavidin-coated magnetic beads were functionalized with an aptamer targeting the receptor-binding domain through a biotin-tagged complementary DNA strand (biotin-cDNA). Upon binding to the receptor-binding domain, the aptamer was released from the biotin-cDNA, allowing the cDNA to initiate RCA on the surface of the magnetic beads. The detection of the receptor-binding domain was accomplished through a dual-signal approach. For fluorescence detection, the RCA products were combined with a dsDNA probe, which was labeled with both a fluorophore and a quencher. The hybridization of RCA products with the dsDNA probe resulted in the separation of the fluorophore and quencher, leading to fluorescence emission (λex = 488 nm, λem = 520 nm) [42].

A method for detecting multiple pathogenic microorganisms is described, utilizing a DNA composite system encapsulating DNA-stabilized silver nanoclusters (AgNCs) and graphene oxide in conjunction with RCA. Initially, two distinct RCA-based DNA composites are constructed, each linked to a probe consisting of DNA-stabilized AgNCs and an ssDNA aptamer specific to two different bacterial pathogens. Graphene oxide is then incorporated into the system to capture the ssDNA aptamers from the DNA composites and act as a selective quencher for the fluorescence of the DNA/AgNCs complex. When the target bacteria are recognized, the ssDNA aptamer binds to the bacteria, causing it to detach from the graphene oxide surface. As a result, the RCA-based DNA composite generates a strong fluorescent signal [43].

**Figure 5:**
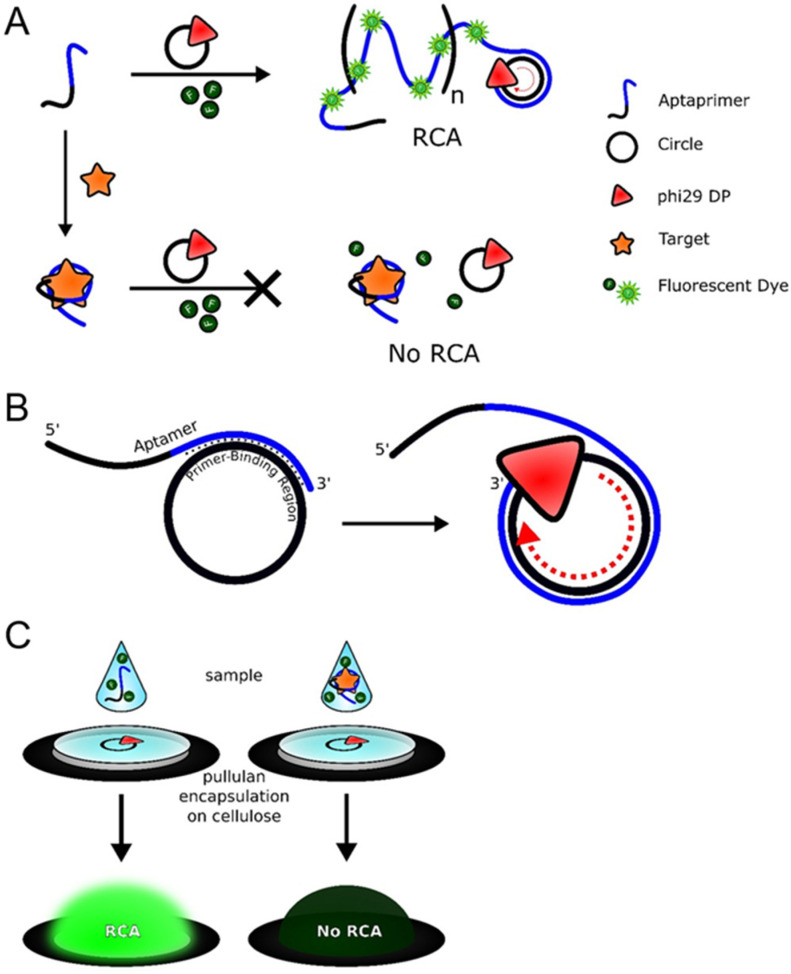
(**A**) Schematic representation of an aptaprimer for target-mediated generation of RCA product by incorporating an intercalating dye such as SYBR Gold or QuantiFluor. The addition of a protein target prevents the binding of the aptaprimer to the circular template, inhibiting RCA. (**B**) Aptaprimer concept: the circle-binding region is embedded within the native aptamer strand on the 3′-end. Polymerization occurs from the 3′-end with phi29DP displacing hybridized aptaprimer to continue elongation of the RCA product. (**C**) A sample mixed with aptaprimer and intercalating dye is spotted onto a wax-contained cellulose well holding pullulan-encapsulated RCA reagents (phi 29DP, circle template, and dNTPs), allowing for real-time one-step RCA and production of a fluorescence signal [37].

Roger et al. present a streamlined approach to integrating protein-binding aptamers with isothermal amplification, enabling a one-step reaction that does not require any labeled or modified DNA species. This method is easily adaptable for use in paper-based point-of-care devices. The assay employs a protein-binding aptamer that also functions as a linear primer, initiating RCA, referred to as an “aptaprimer”. In the absence of a target, the aptaprimer binds to a complementary circle template and triggers RCA in the presence of phi29 DNA polymerase and dNTPs, producing a long DNA product. This product can bind fluorescent dyes like SYBR Gold™ or QuantiFluor™ to generate a fluorescence signal (Figure 5A). When a protein target is introduced, the aptaprimer forms an aptamer–protein complex, which prevents the aptaprimer from binding to the circular template, thereby inhibiting the RCA process and modulating the fluorescence output. In the AP, the primer-binding region is incorporated within the native aptamer at the 3′ end. Polymerization of the AP strand occurs at the 3′ end, and the hybridized AP is displaced by f29DP as it proceeds through the circular template (Figure 5B). The scientists demonstrate the application of this system for detecting two proteins—platelet-derived growth factor and thrombin—showcasing the potential to couple protein-mediated isothermal DNA amplification with a detectable output signal in a simple, one-step assay format. This system can be employed either in a solution or as part of a paper-based point-of-care test (Figure 5C) [37].

### 5.2. Microfluidic Systems

In recent years, aptamer-based microfluidic platforms have seen rapid development, with a growing focus on improving their detection sensitivity. A study presents an innovative RCA approach for highly sensitive whole-cell detection using microfluidic devices [44]. The dual-RCA method comprises two components: a capturing RCA reaction and a signaling RCA reaction [45]. The capturing RCA reaction involves modifying the microfluidic channel surfaces with long, tandem repeating aptamers (poly-aptamers), which effectively capture target *E. coli* O157:H7 cells. Scientists show that poly-aptamer-modified microchannels capture three times as many target cells compared to those modified with mono-aptamers.

In addition, the signaling RCA method is integrated into the dual-RCA approach to significantly amplify the detection signal. The findings reveal that the signal is enhanced by up to 50 times when signaling RCA is used, compared to traditional single fluorescence probes. When both capturing RCA and signaling RCA are combined in one system, the detection signal is further amplified by approximately 250 times. In conclusion, this microfluidic platform, combined with the dual-RCA approach, offers a simple yet promising solution for sensitive whole-cell detection, making it highly suitable for food safety testing [44].

**Figure 6:**
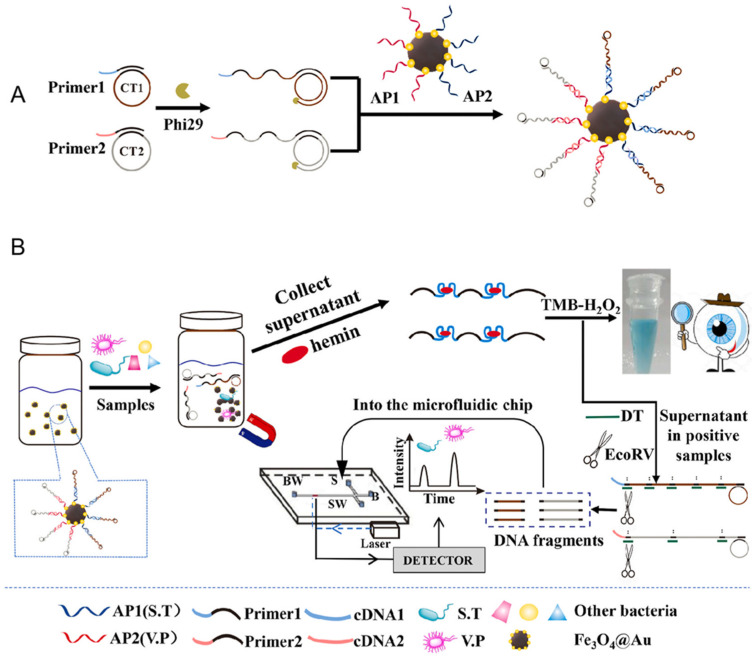
(**A**) The illustration of the preparation of magnetic DNA-encoded probes. RCA produces were linked on magnetic bead surface (circle template 1, 2 (CT1, CT2), 3,3′,5,5′-Tetramethylbenzidine-hydrogen peroxide (TMB-H_2_O_2_)). (**B**) The dual-mode aptasensor for judging the presence and simultaneous determination of S.T and V.P based on RCA reaction in a microfluidic chip. In the presence of S.T. and V.P., they are captured by magnetic DNA-encoded probes to produce a visual signal (target DNA (DT), *Escherichia coli* RY13 V (EcoRV)) [46].

In the study by Yu et al., a dual-mode aptasensor was developed that combines colorimetric detection with a microfluidic chip to achieve the desired outcomes [46]. This aptasensor enables rapid on-site screening that can be visually assessed while also allowing for the simultaneous quantification of multiple bacterial species. Specifically, the presence of pathogenic bacteria, such as Salmonella typhimurium (S.T.) and Vibrio parahaemolyticus (V.P.), was initially detected by visual inspection. Subsequently, the microfluidic chip was used to quantify the levels of S.T. and V.P. in positive samples. To generate the necessary detection signals, a set of magnetic DNA-encoded probes was created (Figure 6A). These probes contained RCA-produced long DNA strands enriched with G-quadruplex sequences. These sequences, in conjunction with hemin, function as DNAzymes that catalyze a reaction with the 3,3′-5,5′-Tetramethylbenzidine-H_2_O_2_ system, producing a colorimetric change. Additionally, the probes were cleaved by EcoRV endonuclease to generate DNA fragments of varying lengths. The microfluidic chip was then used to separate and quantify these DNA fragments, enabling the simultaneous quantification of S.T and V.P (Figure 6B). Using this method, as few as 100 CFU/mL of either V.P. or S.T. could be detected visually, while the microfluidic chip allowed detection down to 32 CFU/mL for S.T. and 30 CFU/mL for V.P. within just 3 min. This dual-mode aptasensor proved to be highly efficient, offering both rapid screening and precise quantification of bacterial contamination in food samples, showcasing its potential for on-site detection and simultaneous quantification of foodborne pathogens.

### 5.3. Visual Detection

Gao et al. developed a dual-mode aptasensor integrating both colorimetric and electrochemical detection, utilizing G4 produced through RCA [47]. During the RCA process, specific binding between the aptamer and target led to the generation of a large number of G4. The colorimetric component of the sensor was based on the interaction between the G4 and hemin, which modulated the 3,3′,5,5′-Tetramethylbenzidine reaction, enabling visual and semi-quantitative detection of kanamycin. For electrochemical detection, the G4 strongly interacted with methylene blue, facilitating the generation of an electrical signal.

This dual-model approach combined the simplicity of colorimetric visualization with the high sensitivity and precision of electrochemical measurements. The aptasensor demonstrated excellent specificity, minimizing potential interference. Additionally, it was effectively applied to monitor kanamycin levels in milk, showing great potential for practical use in food safety and monitoring applications.

**Figure 7:**
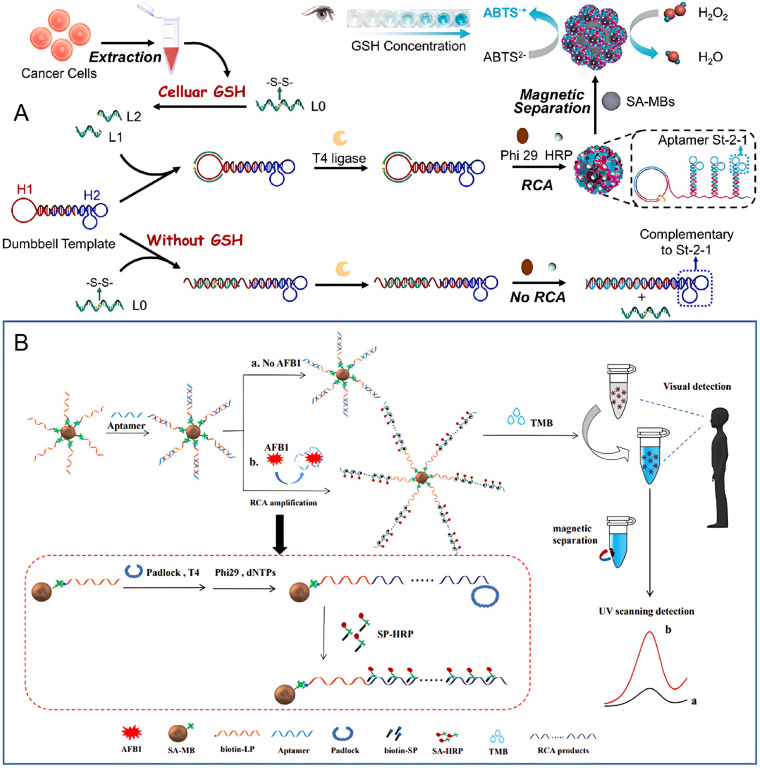
(**A**) Schematic illustration of disulfide cleavage-induced visual biosensing of glutathione based on the dumbbell DNA-mediated RCA for self-assembly of horseradish peroxidase–DNA flower structures using magnetic separation to improve sensitivity (glutathione (GSH), horseradish peroxidase (HRP), streptavidin-magnetic bead (SA-MB), 2, 2′-azino-bis(3-ethylbenzothiazoline-6-sulfonic acid (ABTS)) [48]. (**B**) Schematic illustration of the aflatoxin B1 detection principle based on the aptamer capture and triggered RCA reaction. In the presence of aflatoxin B1, the RCA reaction was triggered and produced a large number of repeated products that can combine with HRP to produce visual signals [49].

In the study by Wang et al., a visual nanoplatform was developed for highly sensitive and precise detection of glutathione using a dumbbell DNA-mediated RCA method [48]. The system consists of a nicked dumbbell probe (H1/H2) and a single-stranded probe (L0). Upon the addition of glutathione, the disulfide bond in the L0 probe is cleaved, producing L1 and L2. These fragments then hybridize with the loop region of H1, initiating the circularization of the dumbbell probe, which serves as a template for RCA (Figure 7A). This triggers the amplification process, during which horseradish peroxidase is encapsulated in three-dimensional DNA flower structures using a one-step method. By converting the detection of unstable glutathione into stable horseradish peroxidase–DNA flower structures with enhanced enzyme activity, this biosensor demonstrates ultra-high sensitivity and accuracy. It successfully detects glutathione in various cancer cell lines, showcasing its potential for precise biomarker analysis.

Zhang et al. developed a highly sensitive and specific visual detection method for aflatoxin B1 that leverages the target selectivity of aptamers, RCA, and enzyme-catalyzed biological amplification [49]. In this approach, the aflatoxin B1-specific aptamer is immobilized onto the surface of magnetic beads, serving as the molecular recognition element. In the absence of aflatoxin B1, the aptamer and the auxiliary linker probe remain in a double-stranded configuration due to partial base pairing. However, in the presence of aflatoxin B1, the aptamer binds selectively to aflatoxin B1, causing the linker probe to return to a single-stranded form. This triggers the RCA process, where the single-stranded linker probe acts as a template to generate long DNA strands. These strands subsequently capture large quantities of signal probes and horseradish peroxidase enzymes. The horseradish peroxidase enzymes then catalyze the oxidation of 3,3′,5,5′-Tetramethylbenzidine by hydrogen peroxide, resulting in a color change from colorless to deep blue, providing a visual signal. This method achieves high sensitivity and specificity for aflatoxin B1 detection (Figure 7B). Furthermore, the aptasensor exhibited remarkable selectivity for aflatoxin B1 when compared to five other common mycotoxins. Notably, all reactions take place on the surface of the magnetic beads, simplifying the detection process. This setup enhances the efficient isolation and collection of aflatoxin B1 from complex sample matrices, improving the sensor’s selectivity and resistance to interference, highlighting its potential for practical application in real-world aflatoxin B1 detection.

Yurdusev group used a model aptamer binding SARS-CoV-2 Spike glycoprotein as a template, triggering the RCA system in a ligand-dependent manner. They confirmed the presence of the Spike protein in the test samples at concentrations within the nanomolar range using an adapted design derived from this model aptamer-RCA system [50].

### 5.4. Electrochemical Sensing Techniques

**Figure 8:**
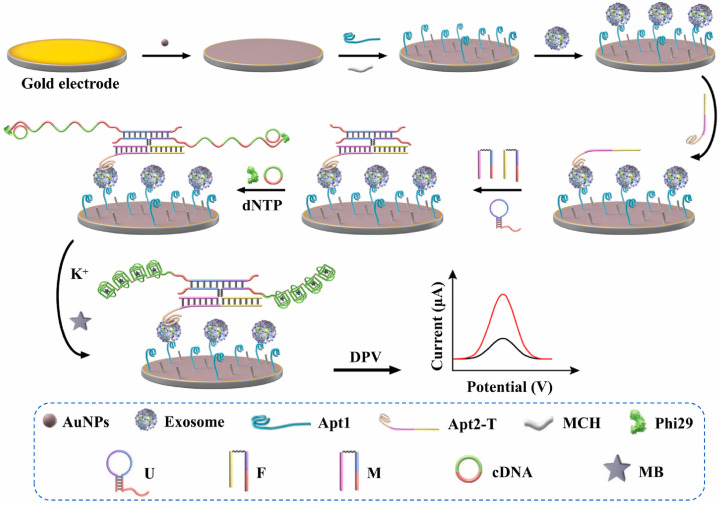
Schematic diagram of a bispecific aptamer sandwich-type AuNPs-modified electrochemical aptasensor for tumor-derived exosome assays based on a four-way junction-triggered dual-RCA-assisted methylene blue/G4 strategy. The repeated G4 structures produced by the RCA reaction were hanging on the electrode surface by the specific binding between split aptamer and exosome, then produced an electrochemical signal to characterize the presence of exosome (6-mercapto-1-hexanol (MCH), strand U (U), strand F (F), strand M (M), methylene blue (MB), differential pulse voltammetry (DPV)) [51].

The accurate quantification of tumor-derived exosomes, which are emerging as highly promising non-invasive biomarkers for cancer diagnosis, is crucial. In response to this need, a novel bispecific-aptamer-based sandwich-type electrochemical aptasensor (Figure 8) was developed [51]. This sensor utilizes gold nanoparticles and incorporates a four-way junction-induced dual RCA-assisted strategy for highly specific and sensitive detection of exosomes. In this design, the aptasensor employs two specific aptamers: one targeting CD63 and the other recognizing the cancer-related protein mucin-1. The CD63 aptamer is immobilized on a gold electrode to capture exosomes, forming the initial complex. When the mucin-1 aptamer is introduced, it binds and completes the sandwich structure. The 3′ end of the MUC1 aptamer facilitates the formation of the four-way junction, aided by a molecular beacon probe and a binary DNA probe. This assembly triggers a dual-RCA reaction, which is initiated when two cytosine-rich circular DNA templates bind to the ends of the four-way junction. The RCA process generates dual-amplified products that contain multiple G4 structures, which in turn trap methylene blue indicators and significantly enhance electrochemical signals. The resulting aptasensor demonstrated exceptional specificity, sensitivity, reproducibility, and stability in detecting exosomes derived from MCF-7 cells. Moreover, this sensor showed excellent potential for use in clinical applications, effectively recovering exosomes from normal human serum samples.

**Figure 9:**
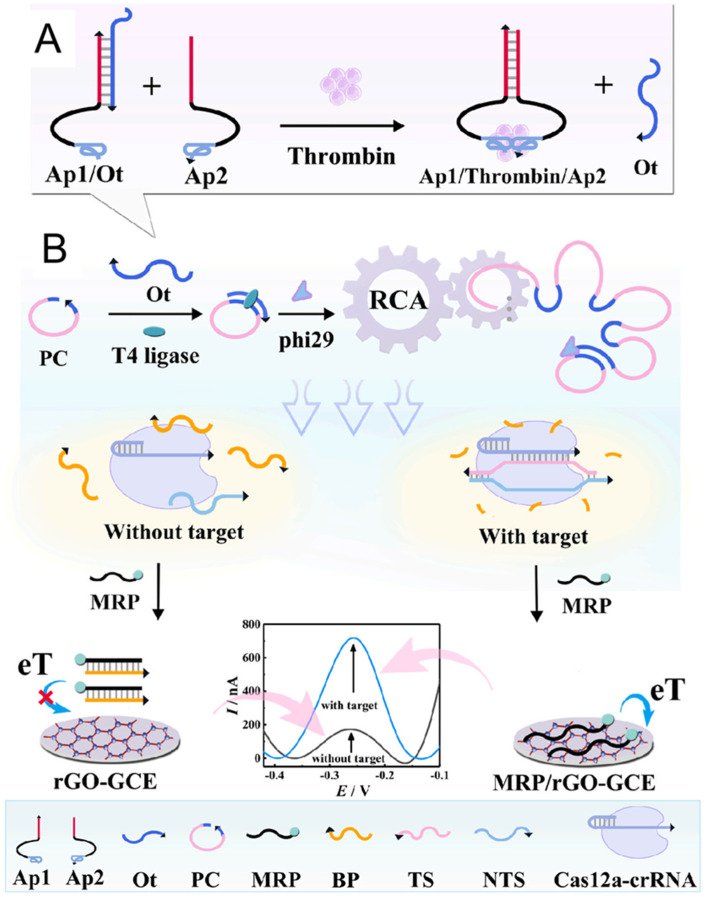
Schematic illustration of the working mechanism of this electrochemical aptasensor for thrombin assay: (**A**) thrombin-triggered binding-induced DNA strand displacement; (**B**) RCA regulated CRISPR/Cas12a system released MRP that can be absorbed on electrode surface to produce an electrochemical signal (output strand (Ot), padlock probe (PC), methylene blue-modified reporter probe (MRP), blocker probe (BP), trigger DNA strand (TS), non-trigger DNA strand (NTS)) [52].

Electrochemical aptasensors are widely employed for detecting and quantifying protein biomarkers, yet their practical use is often constrained by ineffective signal amplification and the complex, time-consuming process of probe surface immobilization. To overcome these challenges, Qing et al. integrated a homogeneous electrochemical aptasensor with the CRISPR/Cas12a system [52]. In their approach, the binding-induced DNA strand displacement (Figure 9A) mechanism is utilized to convert the interaction between thrombin and its aptamer into a nucleic acid signal, which then activates RCA to regulate the nuclease activity of CRISPR/Cas12a (Figure 9B). By exploiting the differential affinities of graphene for single-stranded versus double-stranded DNA, the electrochemical aptasensor eliminates the need for probe immobilization, simplifying the detection process and enhancing its versatility. The combination of binding-induced DNA strand displacement with RCA and CRISPR/Cas12a amplification techniques enables this aptasensor to achieve highly specific and sensitive detection of thrombin, with a limit of detection as low as 1.26 fM. This approach paves the way for more precise and efficient protein biomarker detection.

An aptasensor is presented for electrochemical determination of OTA by the Abnous group based on non-target-triggered production of RCA. The surface of the gold electrode is modified with a thiolated complementary strand of aptamer as both the capture probe and primer and OTA aptamer as both the sensing molecule and padlock probe. Following the addition of OTA, aptamer/OTA conjugate is formed and detached from the electrode surface. Therefore, no RCA is produced after incubation of the modified electrode with T4 DNA ligase and phi29 DNA polymerase, and a sharp current signal occurs. The analytical response ranged from 30 pM to 120 nM with a detection limit of 5 pM [53].

As shown in Table 1, combined with the elaboration about aptamer-RCA biosensing systems, we concluded the features, advantages, and limitations of the four detection methods.

## 6. Summary and Outlook

The highly efficient amplification characteristics of RCA make it a powerful tool for enhancing the sensitivity and specificity of biosensors, particularly in the detection of low-abundance target molecules such as viral RNAs (e.g., SARS-CoV-2 RNA), small-molecule toxins (e.g., OTA, aflatoxin B1), and cell surface markers (e.g., CD63 on exosomes). Aptamers, as highly specific synthetic nucleic acid molecules, can selectively recognize diverse targets including proteins (e.g., thrombin, mucin-1, platelet-derived growth factor), metabolites (e.g., ATP, glutathione), and whole cells or pathogens (e.g., *E. coli*, Salmonella typhimurium), thereby effectively initiating RCA for signal amplification and enabling efficient recognition and quantification of the target analytes (As shown in Table 2).

Currently, the integration of RCA with aptamers has been widely applied in the development of various sensors, particularly in areas such as food safety, environmental monitoring, and disease diagnostics. These sensors offer not only exceptional sensitivity and specificity but also the capability to perform precise detection in complex samples. However, several challenges remain. First, the selectivity of aptamers may be compromised in complex biological matrices where structurally similar molecules (e.g., serum proteins, endogenous nucleases) coexist with the intended targets. Second, RCA reactions may suffer from instability or reduced efficiency under suboptimal conditions, such as low magnesium ion concentration or the presence of polymerase inhibitors. Third, interference from non-target molecules—such as non-specific DNA-binding proteins, competing nucleic acids, or serum albumin—can affect both aptamer binding and amplification fidelity, especially in clinical or environmental samples. Addressing these limitations will require multidisciplinary efforts, including the development of chemically modified aptamers with enhanced binding affinity and nuclease resistance; optimization of RCA polymerase systems for robustness in biological fluids; and integration with orthogonal technologies such as CRISPR/Cas-based detection, nanomaterial-enhanced signal readouts, and microfluidic point-of-care devices. While the integration of aptamers and RCA has demonstrated promising diagnostic capabilities, critical challenges remain. Many current platforms rely on proof-of-concept systems, and their transition to clinical or field use is hindered by complexity, stability concerns, and a lack of standardization across platforms. In particular, trade-offs between sensitivity and operational simplicity must be carefully managed in future research.

Looking ahead, the integration of RCA and aptamers holds great potential to revolutionize next-generation biosensors, particularly in making molecular diagnostics more rapid, accessible, and application-oriented. However, realizing this potential requires targeted advancements across several fronts. First, improving the stability and performance of aptamers in real-world samples remains a key challenge. In clinical or environmental samples, aptamers often face interference from abundant biomolecules such as serum proteins or nucleases. To address this, future efforts may focus on designing chemically modified aptamers that exhibit higher binding affinity, improved structural stability, and resistance to enzymatic degradation. Second, enhancing the robustness of RCA under physiological conditions is essential for its practical use outside of controlled laboratory settings. The development of engineered DNA polymerases with improved tolerance to inhibitors and low ionic strength, or even enzyme-free RCA alternatives using DNAzymes, could help achieve more reliable signal amplification in complex matrices like blood, urine, or food extracts. Third, significant progress can be made by integrating RCA-based biosensors with advanced signal transduction and device platforms, such as the following: Nanomaterials such as gold nanoparticles, carbon nanotubes, and graphene oxide can enhance signal output via fluorescence quenching or electrochemical amplification. Microfluidic chips can automate sample processing and reaction steps, making the entire detection process faster and more user-friendly. Portable devices, including smartphone-based readout systems, can enable point-of-care applications for infectious disease screening or food contamination detection in resource-limited settings. Moreover, RCA-aptamer platforms could be extended to real-time and multiplexed detection. For example, RCA circuits could be engineered to encode unique fluorescent barcodes, allowing simultaneous detection of multiple biomarkers from a single sample, which is a capability crucial for early disease diagnosis and personalized medicine. Lastly, with the growing interest in single-cell analysis, there is great potential to adapt aptamer–RCA strategies for detecting cell-specific surface markers or intracellular molecules at the individual cell level. This would open up new opportunities in cancer diagnostics, immunophenotyping, and stem cell research.

## Figures and Tables

**Table 1:** Comparative summary of different aptamer-RCA biosensing platforms.

Detection Method	Sensitivity	Complexity	Instrument Requirement	Key Advantages	Limitations
Fluorescent	High (fM–pM)	Medium	Fluorescence reader	High sensitivity; visual readout; multiplexing	Prone to background noise; high equipment cost
Electrochemical	Very high (aM–fM)	High	Potentiostat	Portable; excellent sensitivity and specificity	Surface modification complexity; reproducibility issues
Visual	Moderate (nM–pM)	Low	None/Basic	Simple; naked-eye detection	Lower sensitivity; often qualitative/semi-quantitative
Microfluidic	High (pM–fM)	High	Microfluidic chip system	Integrated, fast, miniaturized	High fabrication cost; needs standardization

**Table 2:** Aptamer–RCA integrated biosensing systems.

Aspect	Description	Examples/Notes
Principle	Combination of aptamer-based target recognition with RCA for signal enhancement	RCA generates long DNA products to amplify detection signals upon target recognition by aptamers
Key targets detected	Viral RNAs Small-molecule toxins Cell surface markers Proteins and metabolites Whole cells or pathogens	SARS-CoV-2 RNA OTA, Aflatoxin B1 CD63 on exosomes Thrombin, Mucin-1, PDGF, ATP, Glutathione *E. coli*, Salmonella typhimurium
Application areas	Disease diagnostics Food safety Environmental monitoring	Effective even in complex biological and environmental samples
Advantages	High sensitivity High specificity Signal amplification without thermal cycling Potential for miniaturization and portability	Aptamers provide target recognition; RCA ensures signal amplification
Challenges	Reduced aptamer selectivity in complex samples Inefficient RCA in suboptimal reaction conditions Interference from non-target biomolecules	Serum proteins or nucleases may bind non-specifically Low Mg^2+^ or inhibitors reduce RCA activity DNA-binding proteins or albumin disrupt performance
Strategies to Overcome Challenges	Chemically modify aptamers for better stability and binding Engineer polymerases for robust RCA in biological fluids Integrate with complementary technologies	CRISPR/Cas detection Nanomaterials (e.g., gold NPs, graphene oxide) Microfluidic or portable systems
Future Directions	Real-time biomarker monitoring Single-cell analysis On-site toxin detection using portable sensors	Emphasis on simplicity, standardization, cost-effectiveness, and clinical applicability

## Data Availability

Data will be made available on request.

## Abbreviations

The following abbreviations are used in this manuscript:

functional nucleic acids	FNA
systematic evolution of ligands by exponential enrichment	SELEX
rolling circle amplification	RCA
capillary electrophoresis–SELEX	CE-SELEX
protein microarray system–SELEX	PMM-SELEX
circular DNA template	CDT
deoxyribonucleoside triphosphates	dNTPs
RNA-cleaving DNAzyme	RCD
ochratoxin A	OTA
G-quadruplex	G4
aptamer probe–hairpin primer probe	APH
time-resolved fluorescence nanoparticles	TRFNPs
silver nanoclusters	AgNCs
Salmonella typhimurium	S.T.
Vibrio parahaemolyticus	V.P.
